# The effects of potato virus Y-derived virus small interfering RNAs of three biologically distinct strains on potato (*Solanum tuberosum)* transcriptome

**DOI:** 10.1186/s12985-017-0803-8

**Published:** 2017-07-17

**Authors:** Lindani Moyo, Shunmugiah V. Ramesh, Madhu Kappagantu, Neena Mitter, Vidyasagar Sathuvalli, Hanu R. Pappu

**Affiliations:** 1 Department of Plant Pathology, Washington State University, Pullman, WA 99164 USA; 2 Molecular Plant Sciences Graduate Program, Washington State University, Pullman, 99164 WA USA; 3 ICAR-Directorate of Soybean Research, Indian Council of Agricultural Research (ICAR), Indore, Madhya Pradesh 452 001 India; 4 The University of Queensland, St. Lucia, QLD 4072 Australia; 5 Hermiston Agricultural R&E Center, Oregon State University, Hermiston, OR USA

**Keywords:** Post-transcriptional gene silencing, Small RNAs, In silico, Potyvirus, *Potato virus Y*, Transcriptome, Plant-virus interactions, vsiRNAs, Target prediction

## Abstract

**Background:**

*Potato virus Y* (PVY) is one of the most economically important pathogen of potato that is present as biologically distinct strains. The virus-derived small interfering RNAs (vsiRNAs) from potato cv. Russet Burbank individually infected with PVY-N, PVY-NTN and PVY-O strains were recently characterized. Plant defense RNA-silencing mechanisms deployed against viruses produce vsiRNAs to degrade homologous viral transcripts. Based on sequence complementarity, the vsiRNAs can potentially degrade host RNA transcripts raising the prospect of vsiRNAs as pathogenicity determinants in virus-host interactions. This study investigated the global effects of PVY vsiRNAs on the host potato transcriptome.

**Methods:**

The strain-specific vsiRNAs of PVY, expressed in high copy number, were analyzed in silico for their proclivity to target potato coding and non-coding RNAs using psRobot and psRNATarget algorithms. Functional annotation of target coding transcripts was carried out to predict physiological effects of the vsiRNAs on the potato cv. Russet Burbank. The downregulation of selected target coding transcripts was further validated using qRT-PCR.

**Results:**

The vsiRNAs derived from biologically distinct strains of PVY displayed diversity in terms of absolute number, copy number and hotspots for siRNAs on their respective genomes. The vsiRNAs populations were derived with a high frequency from 6 K1, P1 and Hc-Pro for PVY-N, P1, Hc-Pro and P3 for PVY-NTN, and P1, 3′ UTR and NIa for PVY-O genomic regions. The number of vsiRNAs that displayed interaction with potato coding transcripts and number of putative coding target transcripts were comparable between PVY-N and PVY-O, and were relatively higher for PVY-NTN. The most abundant target non-coding RNA transcripts for the strain specific PVY-derived vsiRNAs were found to be MIR821, 28S rRNA,18S rRNA, snoR71, tRNA-Met and U5. Functional annotation and qRT-PCR validation suggested that the vsiRNAs target genes involved in plant hormone signaling, genetic information processing, plant-pathogen interactions, plant defense and stress response processes in potato.

**Conclusions:**

The findings suggested that the PVY-derived vsiRNAs could act as a pathogenicity determinant and as a counter-defense strategy to host RNA silencing in PVY-potato interactions. The broad range of host genes targeted by PVY vsiRNAs in infected potato suggests a diverse role for vsiRNAs that includes suppression of host stress responses and developmental processes. The interactome scenario is the first report on the interaction between one of the most important *Potyvirus* genome-derived siRNAs and the potato transcripts.

**Electronic supplementary material:**

The online version of this article (doi:10.1186/s12985-017-0803-8) contains supplementary material, which is available to authorized users.

## Background

*Potato virus Y* (PVY) continues to be an economically important pathogen of potato (*Solanum tuberosum* L.), worldwide, that causes significant yield losses and is detrimental to quality of tubers [1–3]. PVY belongs to the family *Potyviridae*, and genus *Potyvirus.* It is a flexuous rod-shaped virus which exhibits a 9.7 kb + ss RNA genome [4]. The genome consists of two opening reading frames which encode 11 proteins. A single large open reading frame encodes a polyprotein cleaved into ten functional proteins while a second small open reading frame called PIPO (Pretty Interesting *Potyiviridae* ORF) encodes P3N-PIPO through RNA polymerase slippage mechanism in the P3-encoding region [2, 4–7].

The genus *Potyvirus* is characterized as one of the largest genera of plant-infecting viruses with more than 200 approved and tentative species of pathogenic viruses [8]. The PVY-potato pathosystem is further complicated by the presence of a complex of different strains of viruses. The viral strains differ from one another in terms of symptoms they produce in same host and expectedly, there exists tremendous amount of genetic variation in their genomic sequences as well as capability for recombination [9–11]. Various strains of PVY infecting potato include ordinary strain (PVY-O), stipple streak strain (PVY-C) and necrotic strains: tobacco veinal-necrotic strain (PVY-N), necrosis tuber-necrotic strain (PVY- NTN), necrotic-wilga (PVY- N:Wi) and a recombinant between N and O (PVY-N:O) [2, 12, 13]. Among the various strains, PVY-O is most prevalent strain in Europe and the USA, along with that, PVY-N and PVY- NTN are the most widely studied strains of PVY. However, recently, an increase in prevalence of recombinant strains, PVY-NTN and PVY-N:Wi over PVY-O has been reported [14]. Potato cultivars show differential host response when challenged with different strains of PVY. A deep sequencing study has revealed the differential accumulation of small RNAs derived from PVY-N, PVY-NTN and PVY-O upon infection of the same potato host cv. Russet Burbank [15].

Plant-pathogenic virus infections usually lead to production of virus-derived small interfering RNAs (vsiRNAs) in infected plant cells, as a result of host RNA silencing-mediated defense mechanism [16–18]. Plant’s defense mechanism is established due to vsiRNAs generated as an outcome of virus infection targeting homologous viral transcripts. The process of formation of dsRNA sequences of virus genome is proposed to occur through various mechanisms like involvement of virus genome-encoded, RNA-dependent RNA polymerases (RdRp), inadvertent complementarity or base pairing between plus and minus strands of viral RNAs, formation of fold-back structures of viral genome sequences due to complementarity or due to the activity of host-derived RdRp etc. [19]. Despite the variation in mechanism, the trigger for RNA silencing is dsRNA which is processed into small interfering RNAs (siRNAs) by the activity of host RNAse III enzymes such as Dicer-like (DCLs). The resultant siRNAs are recruited on to RNA-induced silencing complex (RISC) mediated by family of proteins called argonautes (AGOs) that cleave target RNA in a sequence dependent manner [20–22].

The RNA silencing process also comprises an amplification of signal strategy wherein host-derived RdRp are involved in generation of perfect dsRNA substrates for processing by DCL. This process leads to production of secondary siRNAs which further reinforces the activity of primary siRNAs [23–25]. The role of secondary siRNAs pertains to the systemic spread of silencing signals throughout the plant system [26, 27]. Thus, the entire mechanism of RNA silencing mainly functions as a molecular antiviral defense system to resist the invasion of plant pathogenic viruses. However, the RNAi-mediated silencing of gene expression functions on the principle of sequence complementarity irrespective of the origin of transcripts. Hence it is conceivable and possible to study for the vsiRNAs to have unintended silencing effect on the host transcripts.

With the increase in small RNAs datasets from deep sequencing studies, there is increased development of tools for prediction and identification of their prospective target genes in biological cells as way of functional characterization [28–31]. The application of small RNA target prediction tools is paramount to the elucidation of cellular, physiological and ecological processes [28–33]. This study exploits small RNA target prediction tools along with other bioinformatics tools to elucidate global virus-host interactions at the transcriptome level for the PVY-potato pathosystem.

In this study, we aimed to identify the derivation of vsiRNAs from the genome of three biologically distinct strains of PVY (PVY-N, PVY-NTN and PVY-O) and to reveal their propensity for potato transcripts for post-transcriptional gene silencing. This deciphered the role of PVY-derived vsiRNAs as potential pathogenicity determinants in interfering with host potato physiological processes, stress responses and subsequent symptom development.

## Methods

The data for the study has not been obtained directly or indirectly from human or animal subjects nor has the study been conducted on human/animal subjects hence the research has been exempted from Washington State University Institutional Review Board’s (WSU-IRB) review.

### vsiRNAs and mapping

From our previous study [15], we obtained the complete potato specific small RNAs including virus-derived small interfering RNAs (vsiRNAs) profile of three biologically distinct PVY strains (PVY-N, PVY-NTN, PVY-O) infecting the potato cv. Russet Burbank. The vsiRNA profiles obtained from the three independent small RNA libraries corresponding to infections from the three strains of PVY were aligned using Bowtie (v1.1.0), allowing for perfect matches only [34]. The references used were the complete genomic sequences of the three PVY strains: O (GenBank accession number, HQ912895.1), N (AY884983.1) and NTN (FJ204166) that were previously published [9, 35, 36].

### Prediction of vsiRNAs target potato transcripts

The complete profile of vsiRNAs was categorized into ‘low’ (<50 reads), ‘high’ (> = 50 reads) and ‘very high expression’ (> = 1000 reads) based on the copy or count numbers. The vsiRNAs reads that were between 21 and 24 nt long and had at least 50 copies aligned to the respective PVY genomes were considered as query vsiRNAs for target transcript prediction analysis.

The vsiRNAs profile was used as a query in psRobot small RNA target prediction algorithm utilizing the following default settings: target penalty score threshold – 2.5, five prime boundary of essential sequence – 2, three prime boundary of essential sequence – 17, maximal number of permitted gaps – 1, position after which with gaps permitted – 17 [29]. Potential target coding transcripts of these vsiRNAs were predicted against *S. tuberosum* SolTub_3.0 cDNA sequences downloaded from Ensembl (ftp://ftp.ensemblgenomes.org/pub/plants/release-34/fasta/solanum_tuberosum/cdna/) [37]. Lists of the most highly-targeted transcripts were defined as those having at least 1000 copies of vsiRNA sequences (across multiple vsiRNAs) predicted to target them. The psRNATarget algorithm (http://plantgrn.noble.org/psRNATarget/) [38] was also used to predict target coding transcripts under the following default settings: number of top targets – 200, expectation – 3, penalty for G:U pair – 0.5, penalty for other mismatches – 1, extra weight in seed region – 1.5, seed region – 2-7 NT, HSP size – 20, allow for bulge (gap) on target (penalty for opening gap – 1, penalty for extending gap – 1), calculate target accessibility (Max UPE – 25, flank length – 17/13 NT), translation inhibition range 9 NT- 11 NT [30]. The psRNA Target algorithm was also used to predict potential non-coding transcript targets in *S. tuberosum* SolTub_3.0 non-coding RNAs (ncRNAs) downloaded from Ensembl (ftp://ftp.ensemblgenomes.org/pub/plants/release-35/fasta/solanum_tuberosum/ncrna/) [39] using the same settings as above. Biovenn (http://www.cmbi.ru.nl/cdd/biovenn/) [40] was used to make comparisons among psRobot predicted target coding transcripts for vsiRNAs of PVY-N, −NTN and -O strains and for psRNATarget predicted target ncRNA transcripts [41]. Comparisons/ overlaps were also made for transcript predictions between psRobot and psRNATarget algorithms.

### GO and KEGG pathway analysis of target coding transcripts

Gene ontology (GO) enrichment analysis of the target coding transcripts from the *S. tuberosum* annotation was calculated using the g:Profiler online tool (http://biit.cs.ut.ee/gprofiler/gconvert.cgi) [42] under the g:GOSt Gene Group Functional Profiling category [43]. Transcript IDs were used as query and the following settings were changed from default for the analysis: Organism - *Solanum tuberosum*, Hierarchical filtering - Best per parent group (strong).

Pathway mapping of the target coding transcripts was performed using the transcripts IDs as query and ‘PGSC_DM_v3.4_cds_nonredundant.fasta’ (http://solanaceae.plantbiology.msu.edu/pgsc_download.shtml) [44] of *S. tuberosum* Group Phureja DM1–3 Genome Annotation v3.4 (based on v3 superscaffolds) [45] as KEGG automatic annotation server (KAAS) (http://www.genome.jp/tools/kaas/) [46] input file [47]. The parameters set for running KAAS were as follows: Organisms- all available plants, using KAAS version automatic annotation server ver.1.69× using the methodology bidirectional best hit. The output files were extracted and manipulated using custom PERL scripts.

### Plant growth, RNA extraction and expression analysis by qRT-PCR

Ten coding transcripts, representing various molecular functions, predicted by both algorithms and found to be commonly targeted by strain-specific vsiRNAs of PVY-N, PVY-NTN and PVY-O were selected for qRT-PCR validation in PVY-NTN infected *S. tuberosum* cv. Russet Burbank. The transcripts were selected based on their predicted target score threshold (0–5, lower score is better) and abundance of vsiRNAs targeting them as predicted by both algorithms.

Total RNA was extracted from leaves of PVY-NTN-inoculated *S. tuberosum* cv. Russet Burbank plants, grown under greenhouse conditions as described by Naveed et al. 2014 [15], using Trizol reagent (Invitrogen, Carlsbad, CA, USA). Extracted RNA was treated with 2 U of DNase I (Ambion, TX, USA) at 37 °C for 30 min. The manufacturers’ instructions, were followed for heat inactivation of DNase I. RNA quality was measured by Nanodrop 1000 spectrophotometer (Thermo Scientific, Whaltham, MA, USA) and integrity checked by agarose gel electrophoresis.

Reverse transcription into cDNA was conducted in a 20 μl reaction of 4 μl of 5X iScript Reverse Transcription Supermix (Biorad, Hercules, CA, USA), 1 μg of RNA and made up to 20 μl with nuclease-free water. The following conditions were used: priming at 25 °C for 5 min, reverse transcription at 42 °C for 30 min and inactivation at 85 °C for 5 min. qRT-PCR was performed in 20 μl reaction containing 10 μl of 2X SsoAdvanced Universal SYBR Green Supermix (Bio-Rad, Hercules, CA, USA), 2 μl of 50 ng cDNA, 0.7 μl (10 μM) of each primer and 6.6 μl of nuclease free water. The primer sequences used for the expression analysis of target transcripts (Additional file 1: Table S1) were designed through Biosearch technologies (https://www.biosearchtech.com/support/tools/design-software/realtimedesign-software) [48]. Previously reported elongation factor 1-alpha (ef1alpha) and actin genes were used for normalization as internal reference [49]. The reaction conditions were carried out in Biorad iQ5 real-time PCR detection system (Biorad, Hercules, CA, USA) as follows: initial polymerase activation and DNA denaturation at 95 °C for 30 s, followed by 45 cycles of denaturation at 95 °C for 15 s and annealing/ extension + plate read at 60 °C for 30 s. This was followed by a melt curve at 55–95 °C at 0.5 °C for 2–5 s/step. The 2^-∆∆Ct^ method was used to determine relative gene expression [50].

## Results

### vsiRNAs population characterization

The total clean 21-24 nt reads obtained from PVY-N library aligned to PVY-N genome were 3,504,410 (26% of all 21-24 nt reads in PVY-N) whereas total clean reads for PVY-NTN and PVY-O libraries were 4,397,244 (41%) and 3,277,411 (28%), respectively (Table 1). Differential accumulation of virus strain-specific siRNAs was thus observed despite the same host/potato cultivar (Russet Burbank).

**Table 1:** Total number of small RNAs reads from PVY-N, PVY-NTN, PVY-O strains mapped to their respective *Potato virus Y* genome

S.No	sRNA library	Reference genome	Total no. of reads aligned	Unique vsiRNAs Mapped
1	PVY-N	PVY-N (AY884983.1)	3,504,410	37,453
2	PVY-NTN	PVY-NTN (FJ204166)	4,397,244	38,053
3	PVY-O	PVY-O (HQ912895.1)	3,277,411	32,086

Virus-derived small interfering RNAs (vsiRNAs) derived from three small RNA (sRNA) independent libraries of *Potato virus Y* (PVY) strains, tobacco veinal-necrotic strain (PVY-N), necrosis tuber-necrotic strain (PVY-NTN) and ordinary strain (PVY-O) in potato cv. Russet Burbank were mapped to their respective reference genome using Bowtie v 1.1.0, allowing for perfect matches only

**Fig. 1:**
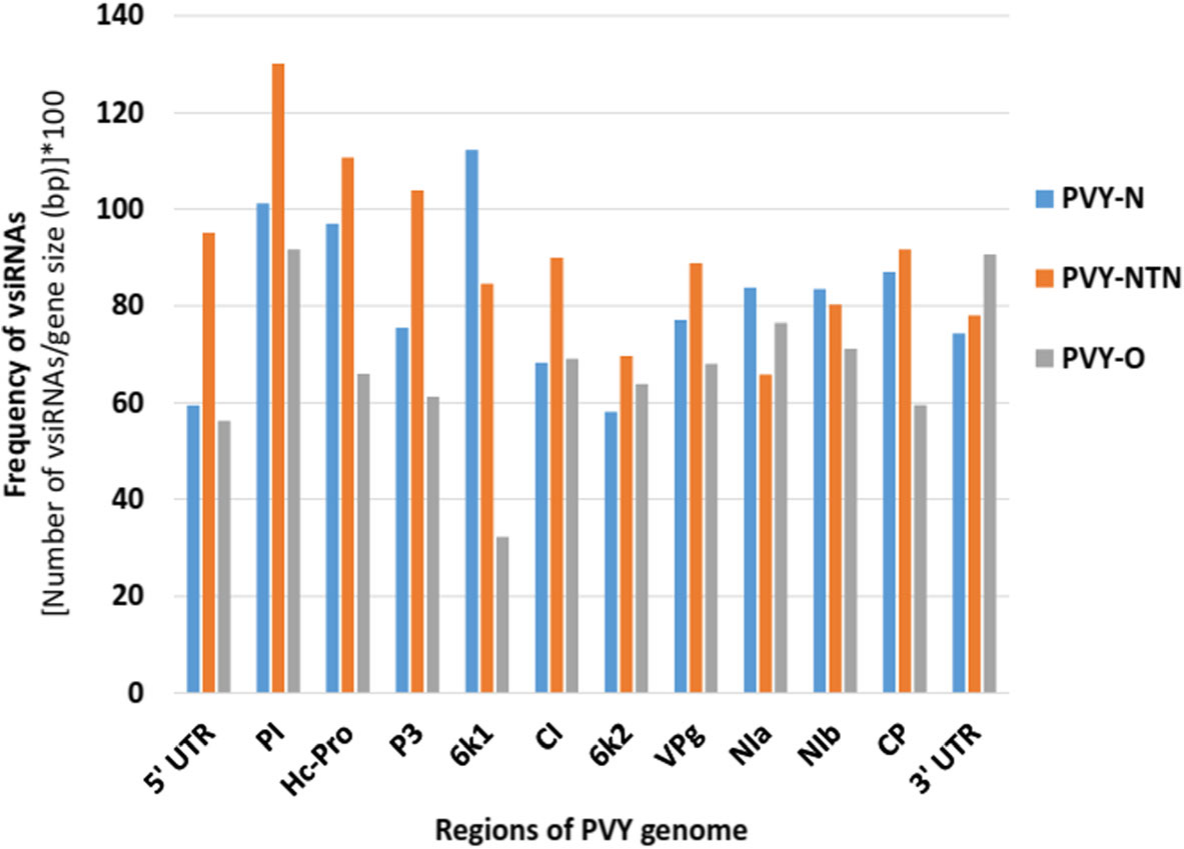
Frequency of small RNA reads in infected potato cv. Russet Burbank plants mapped to respective individual genes of *Potato virus Y* (PVY) using bowtie (v1.1.0). Small RNA reads mapped to PVY genome of tobacco veinal-necrotic strain (PVY-N) are shown in blue. Small RNA reads mapped to PVY genome of necrosis tuber-necrotic strain (PVY-NTN) are shown in orange. Small RNA reads mapped to PVY genome of ordinary strain (PVY-O) are shown in grey

In total, 37,453, 38,053 and 32,086 unique vsiRNAs were mapped to the genome (including UTRs) of PVY strains PVY-N, PVY-NTN and PVY-O, respectively. When unique reads were mapped to respective individual genes of PVY genomes, it revealed considerable differences in the population of small RNAs originating from various genomic positions of the PVY genome. More so, the vsiRNAs exhibited strand bias toward the sense strand of the virus genome over the antisense strand (Additional file 2: Figures S1-S4). The ratio of vsiRNAs in the sense to antisense strands for the libraries of PVY-N, PVY-NTN and PVY-O were 1.5:1, 1.3:1 and 1.6:1, respectively. In case of the N strain, the highest frequency of vsiRNAs generation was shown for 6 K1, PI, Hc-Pro, CP, NIa and NIb genes in descending order, while PI, Hc-Pro, P3, 5′ UTR and CP had the highest frequency for PVY-NTN (Fig. 1). In case of the O strain, the highest frequency of vsiRNAs generation was noticed for PI, 3′ UTR, NIa, NIb and Cl genes (Fig. 1).

### Target potato coding transcripts of PVY-derived vsiRNAs

**Fig. 2:**
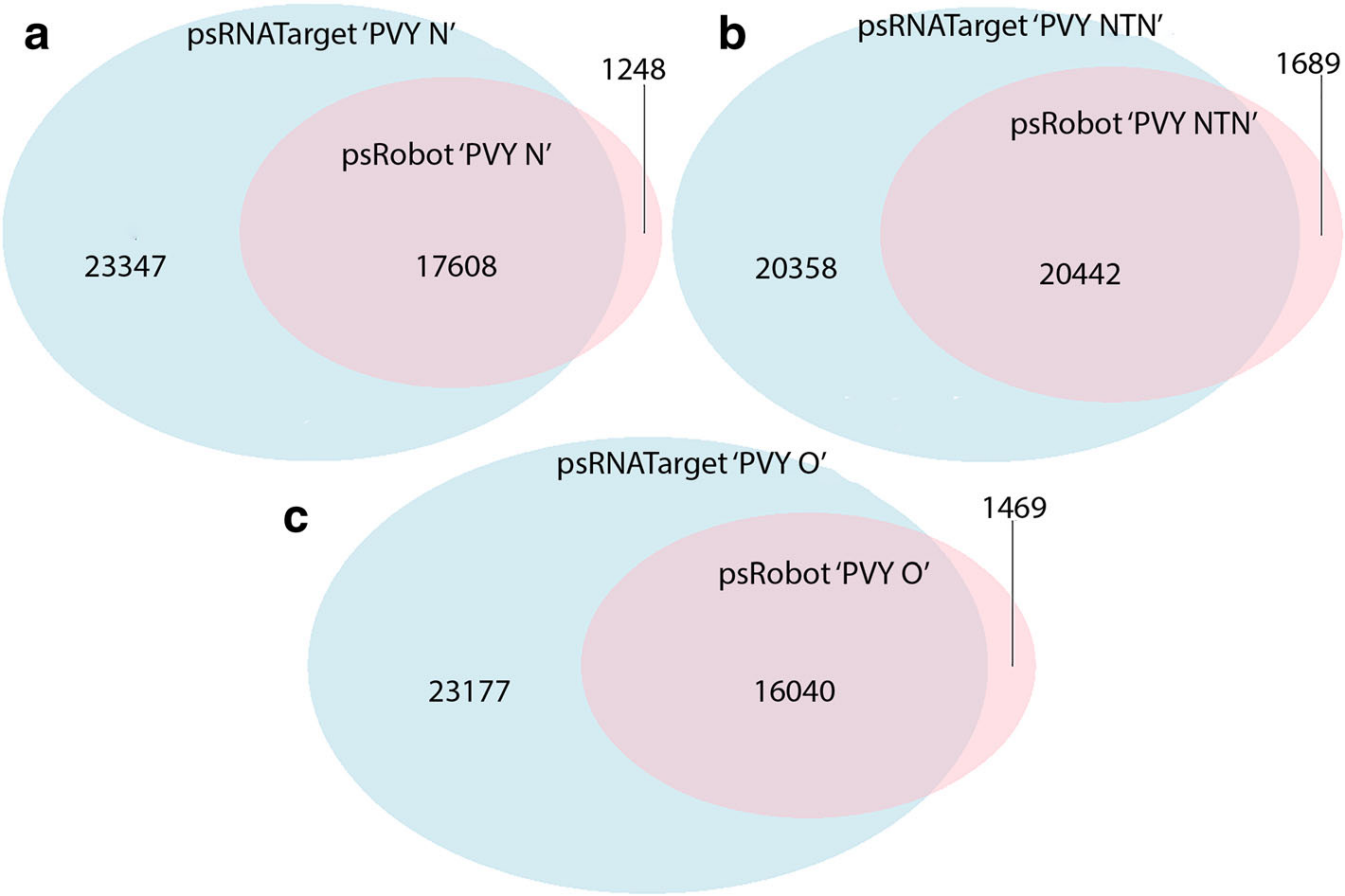
Target potato coding transcripts of *Potato virus Y* (PVY)-derived small RNAs (vsiRNAs) predicted by psRobot and psRNATarget algorithms. Overlaps indicate coding transcripts that were commonly predicted by both algorithms under default settings. **a** Comparison of predicted potato coding transcripts for vsiRNAs of tobacco veinal-necrotic strain (PVY-N) indicated that 93.4% of psRobot algorithm predicted target transcripts were represented in psRNATarget algorithm predictions. **b** Comparison of predicted potato coding transcripts for vsiRNAs of necrosis tuber-necrotic strain (PVY-NTN) indicated that 92.4% of psRobot algorithm predicted target transcripts were represented in psRNATarget algorithm predictions. **c** Comparison of predicted potato coding transcripts for vsiRNAs of ordinary strain (PVY-O) indicated that 91.6% of psRobot algorithm predicted target transcripts were represented in psRNATarget algorithm predictions

A total of 47,743 potato coding transcripts exhibited the propensity for downregulation by PVY-N-derived vsiRNAs (Table 2). In total, 5765 unique vsiRNAs had cross-reacting capability with 18,856 unique transcripts (Additional file 3: Table S2). Similarly, a total of 60,513 potato coding transcripts had propensity for downregulation by PVY-NTN-derived vsiRNAs (Table 2). In total, 7590 unique vsiRNAs were revealed to have cross-reacting capability with 22,131 unique transcripts (Additional file 4: Table S3). For the PVY-O strain, a total of 41,254 potato coding transcripts had susceptibility for the PVY-O-derived vsiRNAs (Table 2). The complete vsiRNAs repertoire of the PVY-O strain revealed that 5569 unique vsiRNAs had cross-reaction potential with 17,509 unique transcripts (Additional file 5: Table S4). A comparison of coding transcripts predicted by psRobot and psRNATarget algorithms revealed that the psRNATarget algorithm predicted a greater number of potato transcripts (Fig. 2a-c). However, the bulk of psRobot predictions (> 90%) were represented in the psRNATarget predictions (Fig. 2a-c) to add credence to in silico predictions used in the study.

**Table 2:** PVY-derived vsiRNAs interaction with potato coding transcripts

S.No	sRNA library	Total target coding transcripts	No. of unique targeting vsiRNAs	No. of unique target coding transcripts
1	PVY-N	47,743	5765	18,856
2	PVY-NTN	60,513	7590	22,131
3	PVY-O	41,254	5569	17,509

High copy number (>50 copies) virus-derived small interfering RNAs (vsiRNAs) of small RNA (sRNA) libraries of *Potato virus Y* (PVY) strains, tobacco veinal necrotic strain (PVY-N), necrosis tuber necrotic strain (PVY-NTN) and ordinary strain (PVY-O) in potato cv. Russet Burbank, were employed as query to predict their cross reacting potential with potato coding transcripts using psRobot algorithm under default settings

The PVY-NTN small RNA library exhibited the highest number of derived vsiRNAs, total coding targets in the potato transcriptome as well as unique target transcripts when compared to PVY-N and PVY-O libraries (Table 2). Further as expected, with lesser number of vsiRNAs repertoire from PVY-O small RNA library, the number of target coding transcripts were also found to be less among all the three strains of PVY under study. However, the target numbers were comparable to those of PVY-N library (Table 2). For all the PVY strain small RNA libraries, some of the predicted transcripts were targeted by multiple vsiRNAs.

**Fig. 3:**
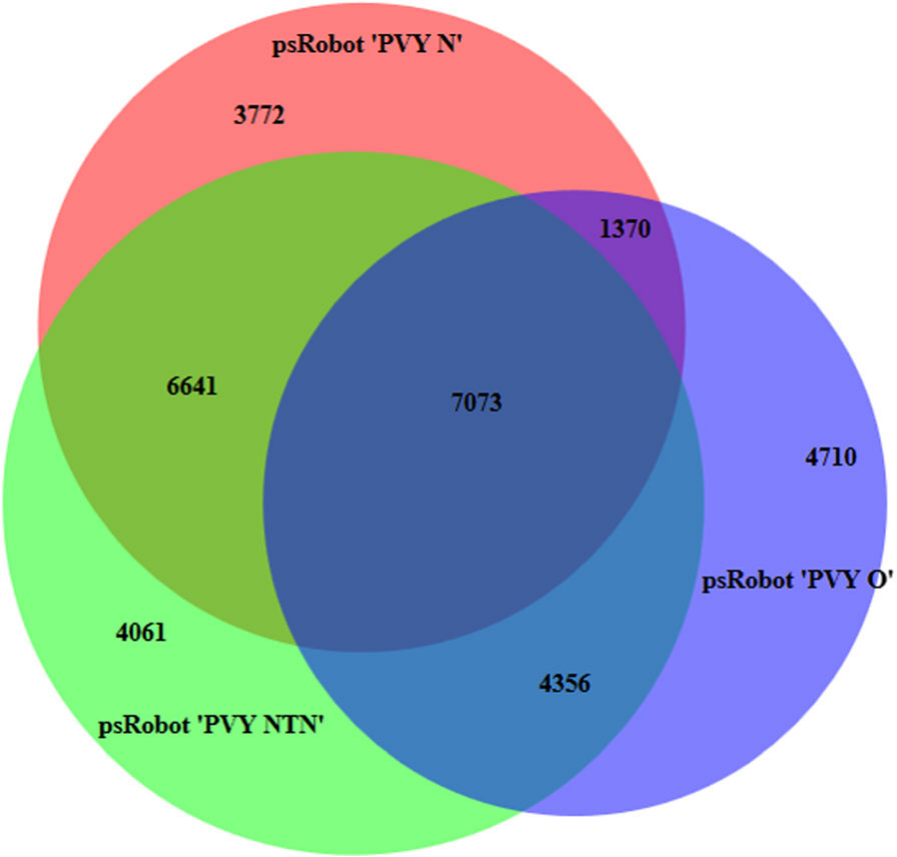
Venn diagram illustrates exclusive and commonly targeted potato coding transcripts predicted by psRobot algorithm for *Potato virus Y* (PVY)-derived small RNAs (vsiRNAs). The illustration shows a comparison of 17,509, 22,131 and 18,856 potato coding transcripts respectively targeted by vsiRNAs of ordinary strain (PVY-O), necrosis tuber-necrotic strain (PVY-NTN) and tobacco veinal-necrotic strain (PVY-N). Transcripts that are commonly targeted by all strains were 7073 (22.1%), while 4710 (14.7%), 4061 (12.7%) and 3772 (11.8%) transcripts were exclusively targeted by vsiRNAs of PVY-O, PVY-NTN and PVY-N, respectively

The comparison amongst psRobot predicted target coding transcripts revealed 7073 (22.1%) transcripts as commonly targeted by vsiRNAs of PVY-O, PVY-N and PVY-NTN (Fig. 3). The vsiRNAs of PVY-NTN and PVY-O, PVY-N and PVY-NTN, PVY-O and PVY-N uniquely targeted 4356 (13.6% of all transcripts), 6641 (20.8% of all transcripts) and 1370 (4.3% of all transcripts) respective coding transcripts out of 11,429 (35.7% of all transcripts), 13,714 (42.9% of all transcripts) and 8443 (26.4% of all transcripts) that were common between them. PVY-O, PVY-NTN and PVY-N exclusively targeted 4710 (14.7%), 4061 (12.7%) and 3772 (11.8%) coding transcripts respectively (Fig. 3).

### Target potato non-coding RNAs (ncRNAs) of PVY-derived vsiRNAs

A total of 2567 potato ncRNAs was predicted to be targeted by 825 unique PVY-N-derived vsiRNAs (Table 3). The 2567 ncRNAs represented 563 unique ncRNA transcripts. For the PVY-NTN library, a total of 1632 ncRNAs representing 401 unique ncRNAs were predicted to be targeted by 870 unique PVY-NTN-derived vsiRNAs (Table 3). A total of 694 unique PVY-O-derived vsiRNAs were predicted to target 2437 ncRNAs representing 485 unique target ncRNAs.

**Table 3:** PVY-derived vsiRNAs interaction with potato ncRNA transcripts

S.No	sRNA library	Total targets in potato ncRNA transcripts	No. of unique targeting vsiRNAs	No. of unique target ncRNA transcripts
1	PVY-N	2567	825	563
2	PVY-NTN	1632	870	401
3	PVY-O	2437	694	485

High copy number (>50 copies) virus-derived small interfering RNAs (vsiRNAs) of small RNA (sRNA) libraries of *Potato virus Y* (PVY) strains, tobacco veinal-necrotic strain (PVY-N), necrosis tuber-necrotic strain (PVY-NTN) and ordinary strain (PVY-O) in potato cv. Russet Burbank, were used as query to predict their cross reacting potential with potato ncRNA transcripts using psRNATarget algorithm under default settings

**Fig. 4:**
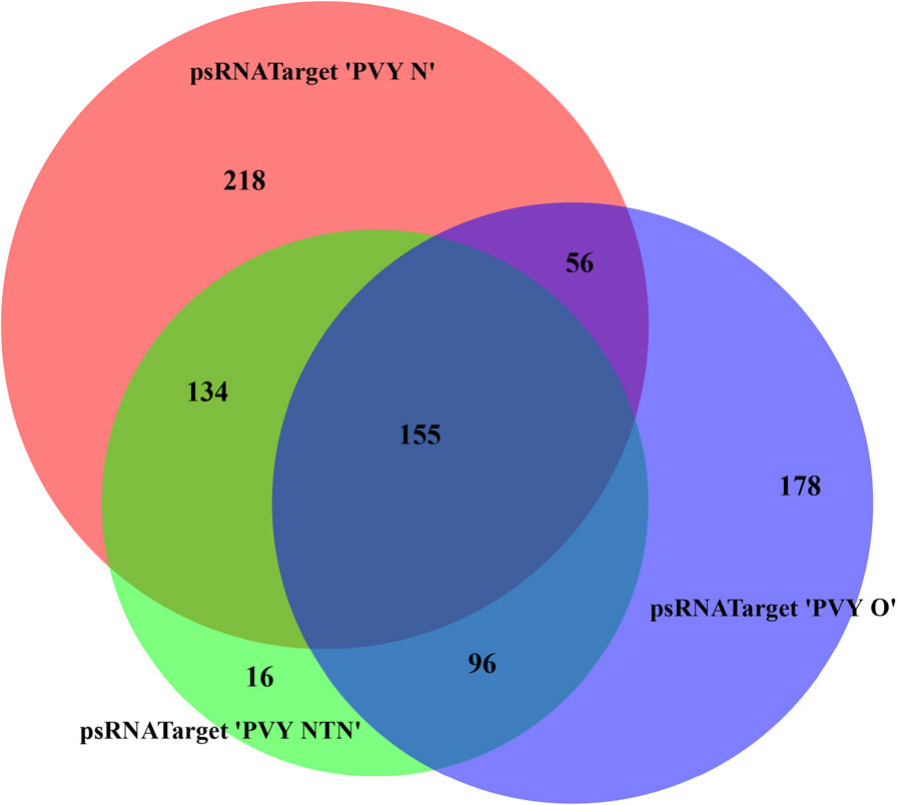
Venn diagram illustrates exclusive and commonly targeted potato ncRNA transcripts predicted by psRNATarget algorithm for *Potato virus Y* (PVY)-derived small RNAs (vsiRNAs). The illustration shows a comparison of 485, 401 and 563 potato ncRNA transcripts respectively targeted by vsiRNAs of ordinary strain (PVY-O), necrosis tuber-necrotic strain (PVY-NTN) and tobacco veinal-necrotic strain (PVY-N). Transcripts that are commonly targeted by all strains were 155 (22.1%), while 178 (20.87%), 16 (1.88%) and 218 (25.56%) transcripts were exclusively targeted by vsiRNAs of PVY-O, PVY-NTN and PVY-N, respectively

The PVY-NTN library exhibited the highest number of unique vsiRNAs targeting the potato ncRNAs, however, they targeted the least number of total and unique ncRNAs compared to the vsiRNAs of PVY-N and PVY-O (Table 3). A total of 155 (18.17%) ncRNAs was shown to be commonly targeted by vsiRNAs of PVY-O, PVY-N and PVY-NTN (Fig. 4). The uniquely targeted transcripts represented 178 (20.87%), 16 (1.88%) and 218 (25.56%) respective ncRNA targets for vsiRNAs of PVY-O, PVY-NTN and PVY-N. For each PVY library of small RNAs, the MIR821 miRNA family was targeted with the highest abundance (Table 4). The abundance of the target ncRNAs, MIR821 was followed by 28S rRNA, 18S rRNA, snoR71, tRNA-Met and U2 amongst others for the PVY-N and PVY-NTN libraries. A similar trend of target ncRNAs abundance was noted for the PVY-O library, however, the MIR821 abundance was followed by U5 and then 28S rRNA, 18S rRNA, snoR71and U2 amongst others (Table 4).

**Table 4:** Abundance of potato ncRNAs targeted by *Potato virus Y*-derived vsiRNAs

PVY-N library		PVY-NTN library		PVY-O library	
Target ncRNA transcript family	Target abundance	Target ncRNA transcript family	Target abundance	Target ncRNA transcript family	Target abundance
MIR821	1292	MIR821	563	MIR821	1309
28S RRNA	344	28S RRNA	370	U5	305
18S RRNA	271	18S RRNA	142	28S RRNA	210
SNOR71	195	SNOR71	122	18S RRNA	206
TRNA-MET	43	TRNA-MET	44	SNOR71	144
U2	39	U2	40	U2	26
SNORD25	29	SNORD25	29	U1	24
MIR-395	27	U1	29	U6	17
U5	22	SNOSNR60_Z15	19	PLANT_U3	16
ACEA_U3	19	MIR-395	19	TRNA-CYS	15
SNOSNR60_Z15	18	SNOR109	15	TRNA-TYR	12
PLANT_U3	18	SNOZ112	15	5.8S_RRNA	9
SNOR137	18	U5	14	SNOR109	9
SNOZ112	15	MIR171_1	13	MIR171_1	9
U6	15	MIR159	12	MIR169_5	8
SNOR44_J54	14	PLANT_U3	11	MIR159	8
MIR159	12	SNOU49	10	SNOR44_J54	6
U1	12	U6	9	SNOR128	6
SNOZ267	11	SNOU19	9	MIR169_2	6
SNOU19	10	SNOZ267	8	SNOR99	6
U3	10	SNORD18	8	SNORD18	5
SNOR69Y	9	SNOF1_F2	8	SNOR126	5
SNOR109	9	TRNA-SER	8	SNOR135	4
SNOR74	9	PLANT_SRP	7	SNORD36	4
METAZOA_SRP	8	SNOR128	7	MIR-172	4
PLANT_SRP	8	MIR169_5	7	SNOR74	4
SNOF1_F2	6	ENOD40	7	MIR-166	4
SNOU49	6	SNOR137	7	SNORD14	4
SNORD14	6	MIR396	6	MIR-598	3
SNOR104	5	MIR-166	6	SNOZ267	3
MIR396	5	SNOR135	5	SNOZ161_228	3
SNOR97	4	SNOR104	5	SNOR11	3
U6ATAC	4	SNOR44_J54	4	MIR-395	3
SNOU30	4	METAZOA_SRP	4	MIR168	3
MIR169_5	4	SNOU30	4	ACEA_U3	3
SNOR103	4	U6ATAC	4	SNOZ196	3
MIR-166	4	MIR169_2	4	SNOZ155	3
MIR-160	3	SNOR126	4	U3	2
SNORD100	3	SNOZ155	3	MIR-160	2
SNOR30	3	TRNA-LEU	2	PK-G12RRNA	2
TRNA-TYR	3	PK-G12RRNA	2	SNOR66	2
ISRR	3	SNOR66	2	SNOJ33	2
TRNA-LEU	3	SNOR116	2	TRNA-PRO	2
ENOD40	2	SNOR24	2	TRNA-MET	2
SNOR135	2	SNOZ196	2	TRNA-LEU	1
SNOR100	2	SNORD100	2	MIR396	1
SNOZ101	2	SNOR30	2	SNORD96	1
5.8S_RRNA	2	SNOR100	2	SNOR116	1
MIR394	2	SNOR60	2	SNOR24	1
MIR169_2	2	SNORD27	2	SNOZ101	1
SNOR21	2	MIR394	2	SNOR114	1
MIR171_1	1	SNOR143	1	TRNA-SER	1
SNOR143	1	SNOR83	1	SNOR104	1
SNOR27	1	SNOR27	1	SNOR32_R81	1
SNOR60	1	MIR-172	1	SNOU30	1
Total	2567	TRNA-TYR	1	Total	2437
		SNOU36A	1		
		SNORD14	1		
		Total	1632		

The abundance of ncRNA targets was grouped by the family or class of the non-coding RNA target transcripts

### KEGG pathway mapping of target coding transcripts

Among the 47,743 target coding transcripts of potato, analyzed against PVY-N vsiRNAs, vast majority of them (42202) could not be assigned to any pathway, however 5541 transcripts were mapped to at least one pathway in KEGG database. Similarly, for the vsiRNAs generated from PVY-NTN and PVY-O libraries, 7172 out of 60,513 and 4624 out of 41,254 targets were mapped to at least one pathway in KEGG database respectively.

**Fig. 5:**
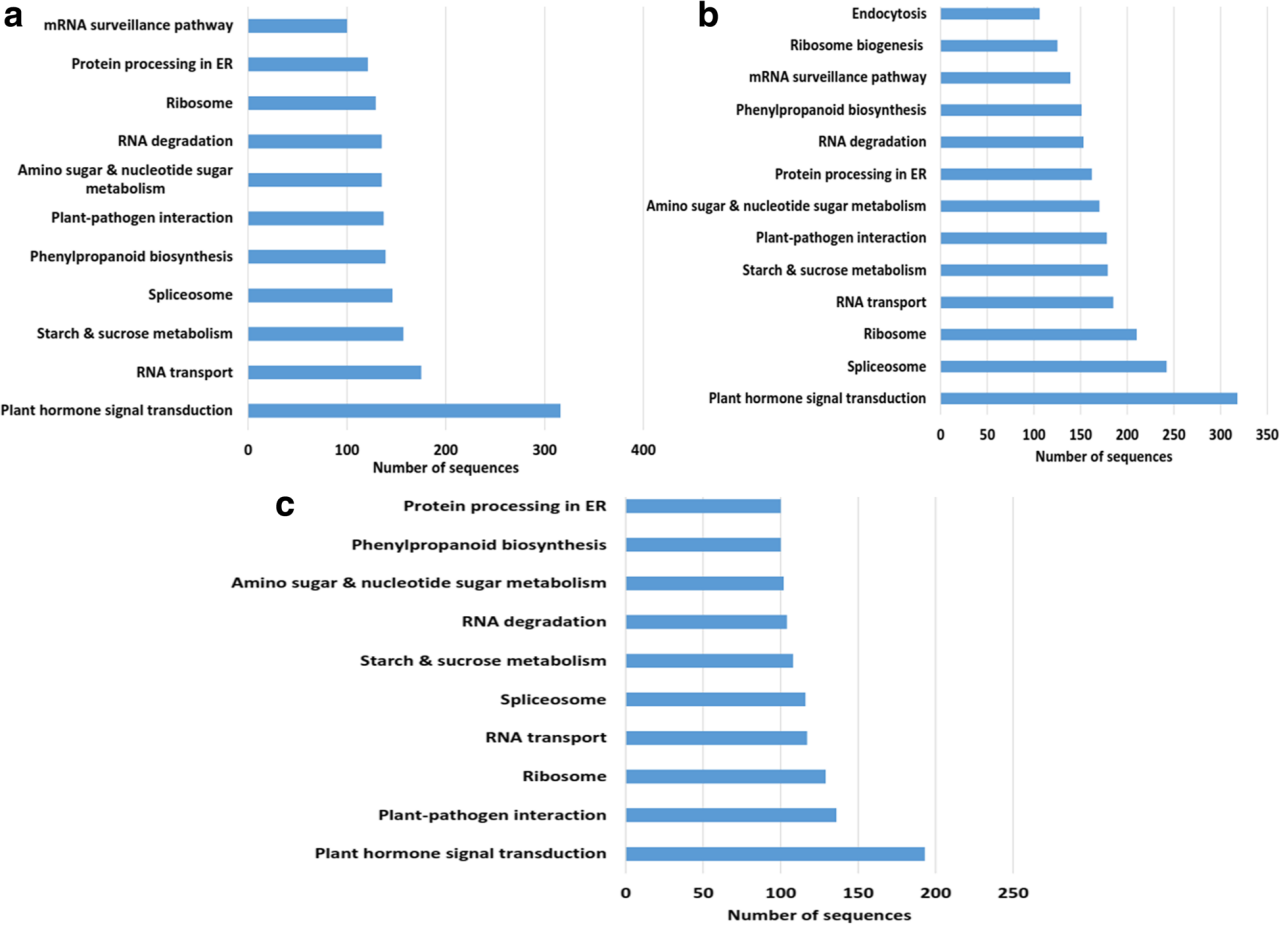
Predicted (psRobot algorithm) target potato coding transcripts annotated to the KEGG pathway. Target pathways by *Potato virus Y* (PVY)-derived small RNAs (vsiRNAs) with minimum of 100 hits are depicted. **a** Tobacco veinal-necrotic strain (PVY-N)-derived vsiRNA targets in potato transcripts annotated to KEGG pathway. **b** Necrosis tuber-necrotic strain (PVY-NTN)-derived vsiRNA targets in potato transcripts annotated to KEGG pathway. **c** Ordinary strain (PVY-O)-derived vsiRNA targets in potato transcripts annotated to KEGG pathway

KEGG pathway enrichment of target coding transcripts of PVY-N library-derived vsiRNAs revealed top target pathways that belonged to plant hormone signal transduction with 316 hits followed by RNA transport (175), starch and sucrose metabolism (157), spliceosome (146) amongst others (Fig. 5a). The PVY-NTN library-derived vsiRNAs exhibited inclination to downregulate coding transcripts involved in plant hormone signal transduction (318) followed by spliceosome (242), ribosome (210), RNA transport (185), starch and sucrose metabolism (179) and plant-pathogen interaction (178) amongst others (Fig. 5b). Similarly, for the PVY-O library-derived vsiRNAs, the top target pathways were found to be plant hormone signal transduction (193), plant-pathogen interaction (136), ribosome (129), RNA transport (117), spliceosome (116), starch and sucrose metabolism (108) amongst others (Fig. 5c). There was similarity among the vsiRNAs generated from three PVY strains in terms of their target pathways.

The general scheme for the vsiRNAs of all the strains entailed targeting plant hormone signaling pathway, genetic information processing pathways like RNA transport, ribosomes, spliceosomes followed by transcripts involved in plant-pathogen interaction (Fig. 5a-c). This suggests that the PVY viral genome-derived siRNAs (vsiRNAs), if they perturb the host transcriptome machinery, it is anticipated that their mode of action would remain the same for all the three strains of PVY. Moreover, they exhibit common set of pathways as targets.

### GO functional analysis of target coding transcripts

**Fig. 6:**
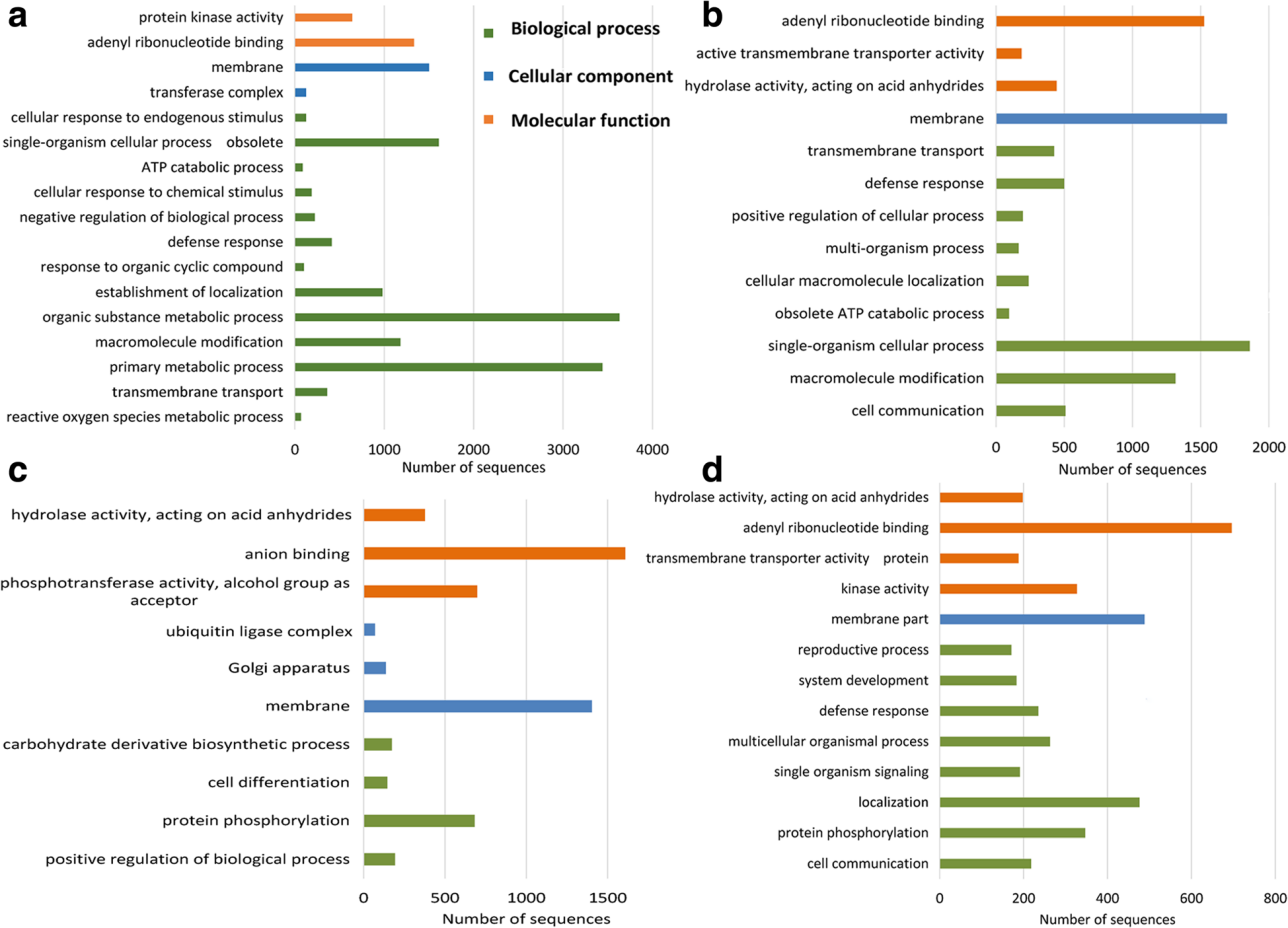
Gene ontology (GO) terms enriched in target potato coding transcripts of small RNAs (vsiRNAs) derived from *Potato virus Y* (PVY) strains, tobacco veinal-necrotic strain (PVY-N), necrosis tuber-necrotic strain (PVY-NTN) and ordinary strain (PVY-O) for the molecular function, biological process and cellular component GO categories. Analysis was conducted using g:Profiler online tool. GO terms with at least 50 hits are depicted. **a** GO terms enriched in target potato transcripts of vsiRNAs derived from PVY-N. **b** GO terms enriched in target potato transcripts of vsiRNAs derived from PVY-NTN. **c** GO terms enriched in target potato transcripts of vsiRNAs derived from PVY-O. **d** GO terms enriched in potato transcripts commonly targeted by vsiRNAs derived from PVY-N, PVY-NTN and PVY-O

The target coding transcripts (Table 2) were analyzed for gene ontology (GO) term studies with the parameters as described in the methods section. The target coding transcripts for vsiRNAs derived from PVY-N strain related to protein kinase activity and adenyl ribonucleotide binding in the molecular function category and to membrane and transferase complex in the cellular component (Fig. 6a). For the biological process, the transcripts related to organic substance metabolic process, primary metabolic process, macromolecule modification, defense response and response to various stimuli (Fig. 6a). Target coding transcripts of PVY-NTN-derived vsiRNAs related to adenyl ribonucleotide binding, hydrolase activity and active transmembrane transporter activity for the molecular function category while they related to the membrane in the cellular component (Fig. 6b). The transcripts related to single-organism cellular process, macromolecule modification, defense response, cell communication amongst others for the biological process (Fig. 6b). GO studies on target coding transcripts for PVY-O-derived vsiRNAs revealed relation to anion binding, phosphotransferase activity and hydrolase activity in the molecular function category while for the cellular component membrane, Golgi apparatus and ubiquitin ligase complex were enriched (Fig. 6c). The biological process was represented by the terms: protein phosphorylation, positive regulation of biological process, carbohydrate derivative biosynthetic process and cell differentiation (Fig. 6c).

The 7073 coding transcripts, that were commonly targeted (Fig. 3) by all the PVY strains, were enriched for membrane part in the cellular component category and for adenyl ribonucleotide binding, kinase activity, hydrolase activity and transmembrane transporter activity in the molecular function category (Fig. 6d). In the biological process, they were enriched for localization, protein phosphorylation, multicellular organismal process, defense response amongst others (Fig. 6d). While no significant enrichment was noted for the 3772 transcripts (Fig. 3) exclusively targeted by vsiRNAs for PVY-N, enrichment was noted for exclusive PVY-O and PVY-NTN vsiRNAs target coding transcripts. The 4710 PVY-O exclusive target transcripts (Fig. 3) were related to phosphorus metabolic process and phosphate-containing compound metabolic process in the biological process category only (data not shown). The 4061 PVY-NTN exclusive target transcripts (Fig. 3) were related to developmental process, anatomical structure development amongst others in the biological process category only (data not shown).

### Validation of PVY-derived vsiRNAs target potato transcripts by qRT-PCR

**Fig. 7:**
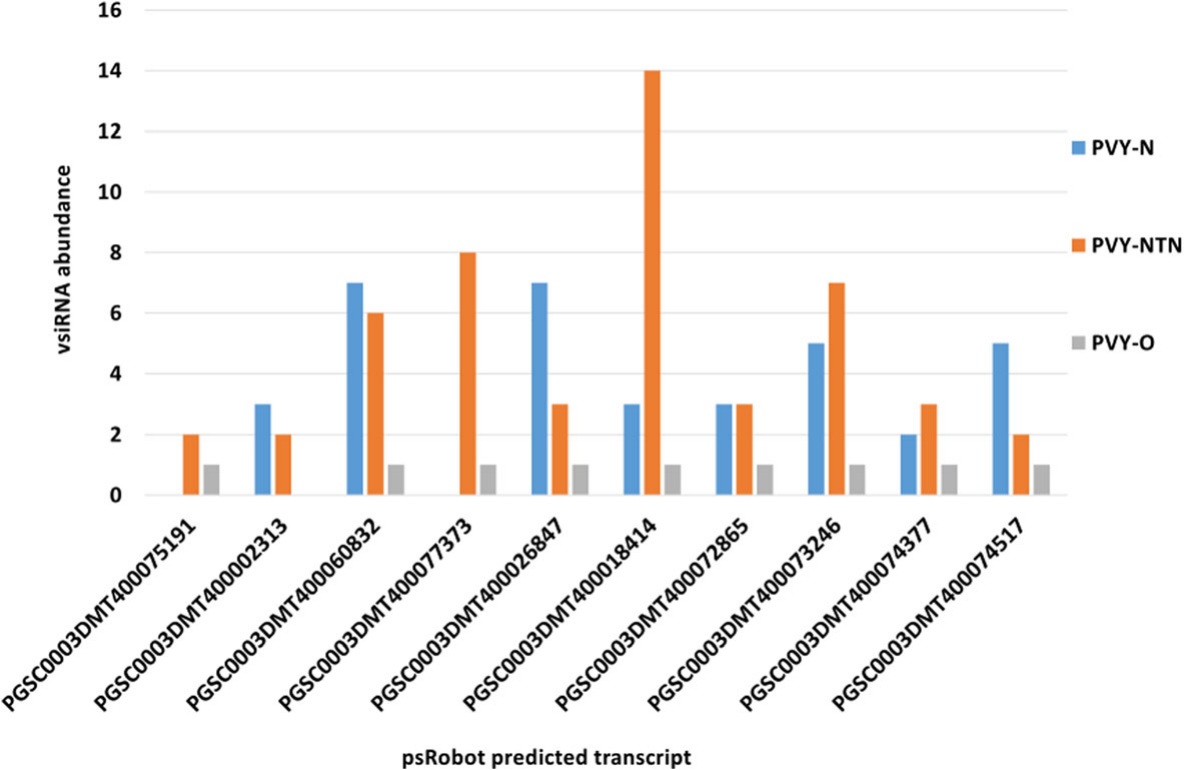
Virus**-**derived small interfering RNA (vsiRNA) abundance for selected target potato coding transcripts. The transcripts were selected from among psRobot algorithm target predictions for vsiRNAs of *Potato virus Y* tobacco veinal-necrotic strain (PVY-N), *Potato virus Y* necrosis tuber-necrotic strain (PVY-NTN) and *Potato virus Y* ordinary strain (PVY-O)

**Fig. 8:**
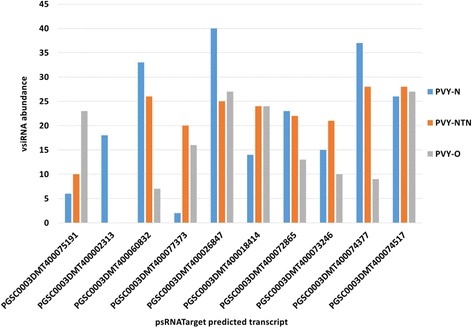
Virus**-**derived small interfering RNA (vsiRNA) abundance for selected target potato coding transcripts. The transcripts were selected from among psRNATarget algorithm target predictions for vsiRNAs of *Potato virus Y* tobacco veinal-necrotic strain (PVY-N), *Potato virus Y* necrosis tuber-necrotic strain (PVY-NTN) and *Potato virus Y* ordinary strain (PVY-O)

Ten coding transcripts were selected for qRT-PCR validation based on their predicted target score threshold (0–5, lower score is better) and abundance of vsiRNAs targeting them as predicted by both algorithms. In psRobot predictions, the transcript PGSC0003DMT400075191 and transcript PGSC0003DMT400077373 (Fig. 7 and Additional file 6: Table S5) were only targeted by vsiRNAs of PVY-NTN and PVY-O, and the transcript PGSC0003DMT400002313 was only targeted by vsiRNAs of PVY-NTN and PVY-N (Fig. 7 and Additional file 6: Table S5). In psRNATarget predictions, the transcript PGSC0003DMT400002313 was only targeted by vsiRNAs of PVY-N (Fig. 8). The psRNATarget algorithm predicted at least one PVY-NTN-derived vsiRNA to target the selected transcripts for cleavage except for the transcript PGSC0003DMT400002313 (data not shown).

**Fig. 9:**
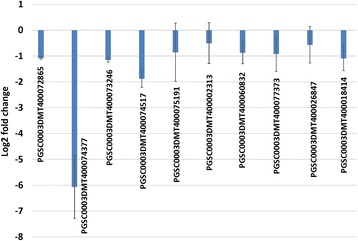
Expression levels of selected target potato coding transcripts commonly targeted by *Potato virus Y* (PVY)-derived small RNAs (vsiRNAs) derived from tobacco veinal-necrotic strain (PVY-N), necrosis tuber-necrotic strain (PVY-NTN) and ordinary strain (PVY-O). In potato plants (Russet Burbank) infected with PVY-NTN, the expression levels of PGSC0003DMT400072865 - protein kinase splA, PGSC0003DMT400074377 - heat shock protein-90 (HSP-90), PGSC0003DMT400073246 - aquaporin NIP1–1, PGSC0003DMT400074517 - β-1,3 galactosyltransferase 2, PGSC0003DMT400075191 - Histone-lysine N methyltransferase, PGSC0003DMT400002313 - BTB/POZ domain containing protein, PGSC0003DMT400060832 - casein kinase, PGSC0003DMT400077373 - Gamma glutamyl transferase 1, PGSC0003DMT400026847 - VPS51/VPS67 family protein and PGSC0003DMT400018414 - phosphoglycerate mutase were downregulated. The elongation factor 1-alpha (ef1alpha) and actin genes were used for normalization. Error bars indicate the standard deviations of qRT-PCR signals (*n* = 3)

Out of the ten coding transcripts targeted in qRT-PCR, two transcripts, PGSC0003DMT400074377 - heat shock protein 90 (HSP-90) and PGSC0003DMT400074517 - β-1,3 galactosyltransferase 2 showed significant change in expression level (*P* < 0.05, Student’s t-test). Transcript expression level of HSP-90 was reduced by log_2_(FC) = −6.07 and β-1,3 galactosyltransferase 2 was reduced by log_2_(FC) = −1.88 (Fig. 9). Interestingly, these transcripts were predicted to be targeted with the highest vsiRNA abundance by psRNATarget for PVY-NTN (Fig. 8). The vsiRNA abundance for these transcripts also ranked amongst the highest for PVY-N predictions, while the vsiRNA abundance for PGSC0003DMT400074517 - β-1,3 galactosyltransferase 2 was among the highest for PVY-O predictions. The transcript expression levels of PGSC0003DMT400072865 - protein kinase splA, PGSC0003DMT400073246 - aquaporin NIP1–1 and PGSC0003DMT400018414 - phosphoglycerate mutase were reduced by log_2_(FC) = −1.08, log_2_(FC) = −1.15 and log_2_(FC) = −1.09, respectively, while for the other transcripts it was log_2_(FC) < 1 (Fig. 9). The transcript, PGSC0003DMT400002313 – BTB/POZ, was not predicted by psRNATarget as a target of PVY-NTN-derived vsiRNAs (Fig. 8) and was predicted by psRobot with vsiRNA abundance of 2 (Fig. 7). It is interesting for this transcript to exhibit the least fold change in qRT-PCR compared to other selected transcripts, thus showing correlation between in silico predictions and qRT-PCR analysis (Fig. 9).

The targeting of heat shock protein (HSP)-90 suggests its potential involvement in plant defense and stress responses [51]. The downregulation of this gene in PVY-NTN infected samples indicates that PVY-derived vsiRNAs could act as pathogenicity determinants. Similarly, the β-1,3 galactosyltransferase 2 is known to be involved in cell wall structure, cell–cell interactions and signaling and host–pathogen interactions. The significant downregulation of these two transcripts in PVY-NTN infected samples adds credence to the functional annotation of target transcripts for their involvement in plant defense, signaling and plant-pathogen interaction pathways. PGSC0003DMT400074377 - HSP-90 and PGSC0003DMT400074517 - β-1,3 galactosyltransferase 2 were predicted as targets of vsiRNAs of PVY-NTN, PVY-O and PVY-N by both psRNATarget (Fig. 8) and psRobot (Fig. 7).

The predicted transcripts that showed no significant change in expression level could also suggest the involvement of other processes or factors in their gene regulation that may include, RNA-silencing suppressors coded for by the virus, abundance and complementarity of vsiRNAs, incorporation of vsiRNAs into the AGOs and their efficiency on mRNA degradation [16].

## Discussion

We studied the role of highly frequent PVY-derived vsiRNAs of three biologically distinct strains of PVY (PVY-N, PVY-NTN and PVY-O) for their putative global effects on the host transcripts. The important resources of small RNA target prediction algorithms, psRobot and psRNATarget, to map vsiRNAs targets on the transcripts of potato and the potato genome annotations (*S. tuberosum* SolTub_3.0 cDNA sequences and *S. tuberosum* SolTub_3.0 non-coding RNAs) were employed effectively to identify genes that are potential targets for vsiRNA-induced silencing. It is vital to note that different small RNA target prediction algorithms predicted target transcripts at varying degrees, as such, intersections in outputs by different algorithms deliver precision and reduces false positives [28]. Large data sets of the potato coding transcripts were predicted to exhibit complementarity with PVY strain-specific vsiRNAs and hence the potential for their downregulation. The targeting vsiRNAs mapped to every genomic position of PVY. The genomic regions of 6 K1, P1 and Hc-Pro in PVY-N, P1, Hc-Pro and P3 in PVY-NTN and P1, 3′ UTR and NIa in PVY-O were dominant hotspots for the generation of vsiRNAs. The high frequency of vsiRNAs substantiates the plausibility of their off-target effects on the host transcripts.

The PVY strains (PVY-N, PVY-NTN and PVY-O) are known to elicit varied biological responses and differential expression of their genome-derived vsiRNAs [2, 15]. With that in mind, we anticipated varied interaction between PVY strain-specific vsiRNAs and the potato transcripts. As such, potato transcripts that were exclusively targeted by each strain may hold important clues about the biological variability and differential response of potato to each PVY strain. Further exploitation of these exclusively targeted transcripts may improve understanding of PVY-potato interactions. Considering off-target silencing, the differential accumulation of PVY-derived vsiRNAs of different PVY strains (Table 1) in the same potato host may also account for differential biological behavior.

The PVY-NTN strain is known to cause tuber necrosis in the potato tubers of susceptible plants and has been associated with manifestation of the potato tuber necrotic ringspot disease (PTNRD) [14, 52, 53]. The disease develops through protrusion of rings on the surface of the potato tuber, these subsequently become sunken and necrotic [52, 53]. While some PVY-N strains have been recorded to exhibit the same phenomena, it has not been reported for PVY-O [52]. It was therefore interesting to note that PVY-NTN, a recombinant strain, exhibited a higher repertoire of PVY-derived vsiRNAs as well as highest number of target coding transcripts. Also, the GO enrichment of PVY-NTN exclusively targeted transcripts which revealed among others, functions related to developmental process, anatomical structure development. It will be conceivable to investigate PVY-NTN exclusively targeted potato transcripts as well as those only common to PVY-N and PVY-NTN for involvement in PTNRD. Earlier studies have revealed that virus or viroid-derived siRNAs are involved in silencing of host genes which in turn leads to development of typical symptoms associated with respective diseases [54–57]. Hence consideration and proof of vsiRNAs as major pathogenicity determinant of disease symptoms in virus-host relationship are not uncommon.

It must be stated that, while some target coding transcripts were exclusive to each strain of PVY, majority of the predicted transcripts were common amongst them (Fig. 3). The same applies for the predicted ncRNAs (Fig. 4). This may be accounted for by the considerable identity between the PVY strains at genomic level [36]. Thus, there could be shared identity among the vsiRNAs of the PVY strains which subsequently had similar targets in the potato transcripts. This suggests a similar mechanism of action of strain-specific PVY-derived vsiRNAs on the potato transcripts. Moreover, this phenomenon is further corroborated by shared KEGG pathways (Fig. 5a-c) and GO terms enriched (Fig. 6a-d) in target potato coding transcripts amongst the PVY strains.

Functional annotation indicated that the target potato transcripts encompassed broad functional properties. The ncRNAs MIR821, 28S rRNA,18S rRNA, snoR71, tRNA-Met and U5 were identified amongst top target candidates for vsiRNAs of PVY. The MIR821 has been shown to be involved in metabolic processes and stress responses [58–61]. The possible involvement of tRNA-Met in translational control in stress has been highlighted [62]. The top target pathways for the PVY strains were plant hormone signaling, genetic information processing pathways (RNA-related processes), plant-pathogen interactions, phenylpropanoid biosynthesis as well as starch-sucrose metabolism amongst others. These pathways represent high level molecular functions that are related to the GO terms enriched for the target transcripts. For example, the high targeting of the GO term related to RNA processing, adenyl ribonucleotide binding (Fig. 6d) relates to KEGG pathways involved in genetic information processing pathways, spliceosome, RNA transport and ribosome (Fig. 5a -c). Enrichment of GO terms related to membrane part, single cell signaling, defense response, protein kinase activity, cell communication and protein phosphorylation (Fig. 6d) suggests targeting of cellular signaling pathways which may be related to the enriched KEGG pathway in plant hormone signaling (Fig. 5a -c). Interference with plant hormone signaling is further revealed by enrichment of GO terms in system development and reproductive process, physiological processes that are most likely to be under the regulation of phytohormones. These phenomena suggest counter-defense strategy by the PVY-derived vsiRNAs to host RNA silencing to perturb plant defense responses and developmental processes, which may ultimately lead to development of symptoms associated with PVY infection. The vsiRNAs could therefore be important determinants in plant-pathogen interactions by mediating post-transcriptional gene regulation of host genes. Furthermore, the off-target silencing of host transcripts is conceivable and potentially enormous since vsiRNAs and target host mRNAs are present in same cellular environment. Nevertheless, at a given point of time not all the transcripts that are potential targets for vsiRNA based repression are present, hence the presence of transcripts and abundance of vsiRNAs, sequence characteristic features of vsiRNAs like 5′ nucleotide etc. are the determinants that could play a major role in off-target silencing activity of vsiRNAs.

In our study, the HSP-90 was identified to be significantly downregulated upon PVY-NTN infection in potato (Fig. 9). The HSP-90 has been demonstrated to be vital in the function of various proteins. It has been shown to be pertinent to stabilize auxin response phenotypes, to influence the loading of small RNAs into argonautes (AGOs) and subsequently the RNA-induced silencing complex and in plant responses to stresses [63–65]. This relates to the GO and KEGG pathway annotations identified in this study. The silencing of HSP-90 suppressed the Pvr9 resistance gene-mediated hypersensitive response in *Nicotiana benthamiana* to *Pepper mottle virus*, a *Potyvirus* [66]. The demonstration of the requirement of HSP-90 for efficient plant response to stress [51] and its importance in host RNA silencing processes [64] highlights the importance of downregulation of potato HSP-90 gene by PVY. In silico predictions have previously demonstrated the HPS-90 as a validated target of vsiRNAs of *Cucumber mosaic virus*, a + ss RNA virus, in tomato [67]. This substantiates the suggestion that the PVY-derived vsiRNAs are a counter-strategy to thwart plant defense processes. This not only validates the in silico prediction, but highlights its importance in elucidating and understanding virus-host interactions.

The β-1,3 galactosyltransferase 2 was also significantly downregulated in PVY-NTN infected potato. Galactosyltransferases are proteins exhibiting membrane-spanning domains, and through glycosylation of glycoconjugates (glycoproteins, glycolipids, and proteoglycans), are involved in a diversity of functions that include cell wall structure, cell–cell interactions and signaling and host–pathogen interactions [68–71]. A β-1,3 galactosyltransferase has been shown to be paramount for pollen development and viability in Arabidopsis [72]. This validates the interference of PVY infection with plant signaling pathways in potato as predicted in functional annotation. The aquaporin NIP1–1 was shown to be downregulated by log_2_(FC) = −1.15 (Fig. 9) in PVY-NTN infected potato plants. Aquaporin proteins are pertinent for water and nutrient transport through living membranes i.e. transmembrane transport, recently, they have also been demonstrated to be involved in carbon dioxide delivery for photosynthesis as well as confer responses to abiotic and biotic stresses [73–76]. This is interesting because it also validates the functional annotation of PVY strain-specific commonly targeted potato transcripts in the cellular component to the membrane part and in the molecular function category to transmembrane transporter activity protein (Fig. 6d). Moreover, the functional annotation of target coding transcripts of PVY-NTN vsiRNAs revealed enrichment of transmembrane transport in the biological process category, membrane in the cellular component category and active transmembrane transporter activity in the molecular function category (Fig. 6b). It has been shown that aquaporin proteins (tonoplast-intrinsic aquaporins) are upregulated in response to biotic stress, furthermore, tomato (*Solanum lycopersicum*) aquaporin transcripts are high in lines resistant to *Tomato yellow leaf curl virus* (TYLCV) as opposed to susceptible lines [77]. This suggests that downregulation of aquaporins and other transmembrane proteins such as the β-1,3 galactosyltransferases may be involved in conditioning the potato plants for susceptibility to PVY.

The findings presented here are in accordance with the earlier works on virus-derived siRNAs and off-target silencing of host transcripts [54, 55, 57, 78, 79]. Similarly, siRNAs derived from TMV (TMV-Cg: Crucifer infecting isolate of TMV) were revealed to target host mRNAs involved in RNA processing and defense response of the host [80]. Off-targeting potential of *Sugarcane mosaic virus* (SCMV)-derived siRNAs with maize mRNAs have been proven and it was revealed that host mRNA involved in ribosome biogenesis, other biotic and abiotic stress-related pathways were the targets [81]. Similarly, tomato target genes (*SolWD40*-repeat) were identified for small RNAs derived from Pospiviroid infecting tomato and downregulation of host mRNAs was demonstrated [79].

Evidence of vsiRNAs interfering with and silencing host plant genes has also been shown [82]. In addition, the credibility for the hypothesis comes from the successful deployment of 21 nt virus genome-derived sequences as effector molecules of silencing in amiRNA-mediated antiviral resistance [83, 84]. It implies that sequence complementarity of 21 nt length with target mRNA is sufficient to induce RNA silencing of cognate transcripts. The predicted interactome scenario is a first report on the interaction between one of the most important *Potyvirus* genome-derived siRNAs and potato transcripts. It highlights the significance of deeper understanding of the role of vsiRNAs on viral replication, pathogenicity and host machinery. These clues on virus-host interactions can be applied in developing novel strategies for disease management.

## Conclusions

The differential accumulation of PVY strain-specific vsiRNAs in potato cv. Russet Burbank was demonstrated. Most of the vsiRNAs populations were derived with high frequency from 6 K1, P1 and Hc-Pro for PVY-N, P1, Hc-Pro and P3 for PVY-NTN and P1, 3′ UTR and NIa for PVY-O genomic regions. The host genes targeted by the PVY-derived vsiRNAs were found to be involved in plant hormone signaling, genetic information processing and defense/ stress responses suggesting a counter-defense strategy by the virus to the host RNA-silencing machinery. The broad range of host genes targeted by PVY-derived vsiRNAs in infected potato suggests a diverse role for vsiRNAs. Future work could focus on the confirmation of the vast majority of the predicted transcripts, more so, on the transcripts exclusively targeted by the PVY strains.

## Additional files

Additional file 1: Table S1. Primers used for qRT-PCR of target transcripts. (DOCX 13 kb)

Additional file 2: Figure S1. Graphical representation of total virus-derived short-interfering RNAs of *Potato virus Y*-NTN, *Potato virus Y*-N and *Potato virus Y*-O mapped to regions of respective *Potato virus Y* genome. **Figure S2.** Graphical representation of total virus-derived short-interfering RNAs originated from sense and antisense strands of *Potato virus Y*-NTN genome mapped to genomic regions of *Potato virus Y*-NTN. **Figure S3.** Graphical representation of total virus-derived short-interfering RNAs originated from sense and antisense strands of *Potato virus Y*-N genome mapped to genomic regions of *Potato virus Y*-N. **Figure S4.** Graphical representation of total virus-derived short-interfering RNAs originated from sense and antisense strands of *Potato virus Y*-O genome mapped to genomic regions of *Potato virus Y*-O. (DOCX 323 kb)

Additional file 3: Table S2. psRobot predicted PVY-N vsiRNAs target coding transcripts. (XLSX 992 kb)

Additional file 4: Table S3. psRobot predicted PVY-NTN vsiRNAs target coding transcripts. (XLSX 1242 kb)

Additional file 5: Table S4. psRobot predicted PVY-O vsiRNAs target coding transcripts. (XLSX 466 kb)

Additional file 6: Table S5. vsiRNA abundance and scores to selected psRobot predicted coding transcripts. (DOCX 16 kb)

## Abbreviations

GO: Gene ontology
KEGG: Kyoto Encyclopedia of Genes and Genomes
ncRNAs: Non-coding RNAs
qRT-PCR: Quantitative reverse transcription PCR
sRNA: Small RNA
vsiRNAs: Virus-derived small interfering RNAs
