# Supplementary material for: The effects of potato virus Y-derived virus small interfering RNAs of three biologically distinct strains on potato (Solanum tuberosum) transcriptome
Source: Virol J. 2017 Jul 17;14:129. doi: 10.1186/s12985-017-0803-8 (PMC5513076; doi:10.1186/s12985-017-0803-8)
Supplement: Supplementary file 1 — Primers used for qRT-PCR of target transcripts. (DOCX 13 kb) [file 12985_2017_803_MOESM1_ESM.docx]

**Additional file 1: Table S1.** Primers used for quantitative real time polymerase chain reaction validation of target coding transcripts.

| ***Solanum tuberosum* transcript id** | **Target transcript name** | **Primers** |
| --- | --- | --- |
| PGSC0003DMT400075191 | Histone-lysine N methyltransferase | **F:** TTCCCTCACCTCATGCTCTTTG  **R:** GCTTCCCTGTGCAGTCATCAG |
| PGSC0003DMT400002313 | BTB/POZ domain-containing protein | **F:** GCAAGCTCACAAAGCGATAC  **R:** CCCTGAGATCGTGAAGGAACA |
| PGSC0003DMT400060832 | Casein kinase | **F:** GTGGTGATGTCCCGAAATGCT  **R:** CCCACCGTCGATGTATTCCT |
| PGSC0003DMT400077373 | gamma-glutamyl transferase-1 | **F:** GGACAGTCGTGACAGGTGAT  **R:** GTCCCTCCAGCAATACTTTGTAG |
| PGSC0003DMT400026847 | Vps51/Vps67 family protein | **F:** GCTTGCCAGACTGACAGAGA  **R:** GGAGCTGACTCATCCTCTAGAAC |
| PGSC0003DMT400018414 | Phosphoglycerate mutase | **F:** GCTGACACAATCACCAAAGTTCA  **R:** TCCACATAGCGTGGGATACAG |
| PGSC0003DMT400072865 | protein kinase splA | **F:** TGGCGTGGAACACCTGTAG  **R:** GGAGCTTCACCAGCAAATTTACCT |
| PGSC0003DMT400073246 | aquaporin NIP1-1 | **F:** CGTGGCAGCTCAAGTGATTG  **R:** GGTGAGGTTCCAACAAAGTGA |
| PGSC0003DMT400074377 | Solanum tuberosum Hsp90-2 | **F:** GAAGCTGGTGAGGATGTTGATGA  **R:** AACGGCATTGAGATGACACCAA |
| PGSC0003DMT400074517 | beta-1,3-galactosyltransferase 2 | **F:** GGACTCGACGTGCTGCATA  **R:** CTGCCTGAGCCTTCCATTCAC |
