# Supplementary material for: The effects of potato virus Y-derived virus small interfering RNAs of three biologically distinct strains on potato (Solanum tuberosum) transcriptome
Source: Virol J. 2017 Jul 17;14:129. doi: 10.1186/s12985-017-0803-8 (PMC5513076; doi:10.1186/s12985-017-0803-8)
Supplement: Supplementary file 2 — Graphical representation of total virus-derived short-interfering RNAs of Potato virus Y-NTN, Potato virus Y-N and Potato virus Y-O mapped to regions of respective Potato virus Y genome. Figure S2. Graphical representation of total virus-derived short-interfering RNAs originated from sense and antisense strands of Potato virus Y-NTN genome mapped to genomic regions of Potato virus Y-NTN. Figure S3. Graphical representation of total virus-derived short-interfering RNAs originated from sense and antisense strands of Potato virus Y-N genome mapped to genomic regions of Potato virus Y-N. Figure S4. Graphical representation of total virus-derived short-interfering RNAs originated from sense and antisense strands of Potato virus Y-O genome mapped to genomic regions of Potato virus Y-O. (DOCX 323 kb) [file 12985_2017_803_MOESM2_ESM.docx]

**Additional file 2: Figure S1.** Graphical representation of total virus-derived short-interfering RNAs of *Potato virus Y*-NTN, *Potato virus Y*-N and *Potato virus Y*-O mapped to regions of respective *Potato virus Y* genome. **Figure S2.** Graphical representation of total virus-derived short-interfering RNAs originated from sense and antisense strands of *Potato virus Y*-NTN genome mapped to genomic regions of *Potato virus Y*-NTN. **Figure S3.** Graphical representation of total virus-derived short-interfering RNAs originated from sense and antisense strands of *Potato virus Y*-N genome mapped to genomic regions of *Potato virus Y*-N. **Figure S4.** Graphical representation of total virus-derived short-interfering RNAs originated from sense and antisense strands of *Potato virus Y*-O genome mapped to genomic regions of *Potato virus Y*-O.


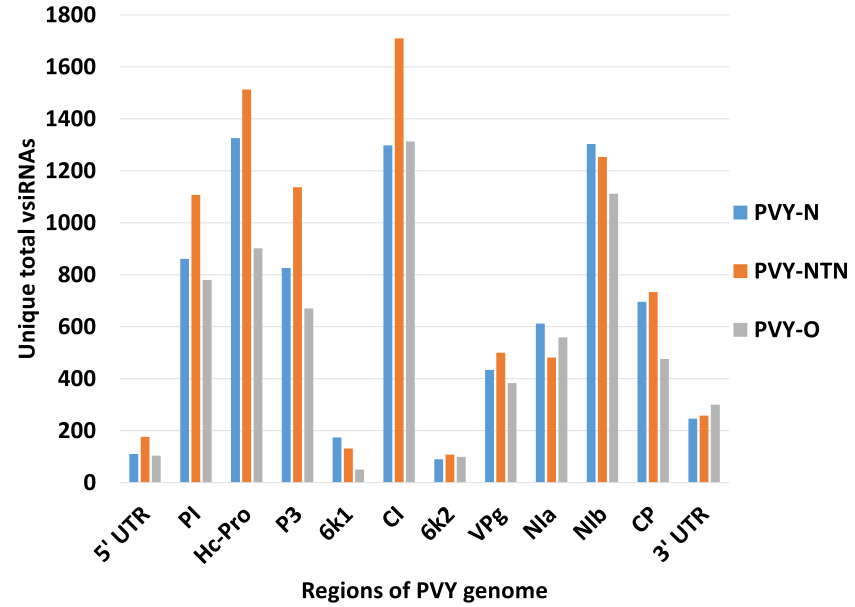


**Figure S1.**


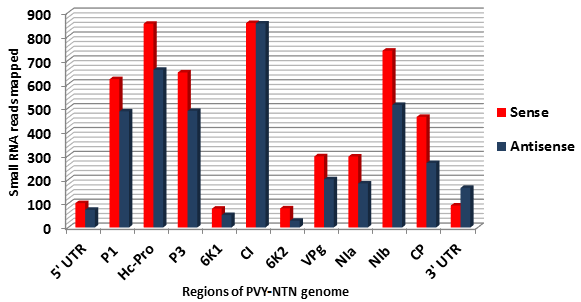


**Figure S2.**


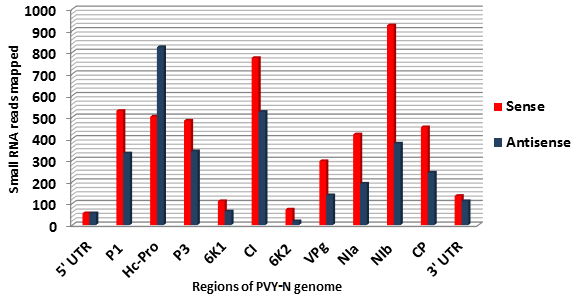


**Figure S3.**


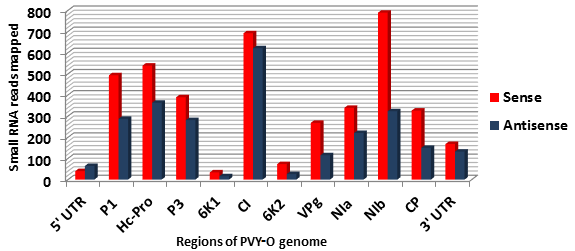


**Figure S4.**
