# Supplementary material for: The effects of potato virus Y-derived virus small interfering RNAs of three biologically distinct strains on potato (Solanum tuberosum) transcriptome
Source: Virol J. 2017 Jul 17;14:129. doi: 10.1186/s12985-017-0803-8 (PMC5513076; doi:10.1186/s12985-017-0803-8)
Supplement: Supplementary file 6 — vsiRNA abundance and scores to selected psRobot predicted coding transcripts. (DOCX 16 kb) [file 12985_2017_803_MOESM6_ESM.docx]

**Additional file 6: Table S5.** vsiRNA abundance and scores to selected psRobot predicted coding transcripts.

| **Solanum tuberosum transcript id** | **Target transcript** | **PVY-N** | | **PVY-NTN** | | **PVY-O** | |
| --- | --- | --- | --- | --- | --- | --- | --- |
|  |  | **vsiRNA abundance** | **Target scores** | **vsiRNA abundance** | **Target scores** | **vsiRNA abundance** | **Target scores** |
| PGSC0003DMT400075191 | Histone-lysine N methyltransferase | 0 | - | 2 | 2.5 (x2) | 1 | 2.5 |
| PGSC0003DMT400002313 | BTB/POZ domain-containing protein | 3 | 0.5  1.0  2.5 | 2 | 2.0  2.5 | 0 | - |
| PGSC0003DMT400060832 | Casein kinase | 7 | 1.0  2.0 (x3)  2.2  2.5 (x2) | 6 | 1.0  2.0  2.5 (x4) | 1 | 2.5 |
| PGSC0003DMT400077373 | gamma-glutamyl transferase-1 | 0 | - | 8 | 0.8  1.0  1.2  1.5  2.0  2.5 (x3) | 1 | 0.8 |
| PGSC0003DMT400026847 | Vps51/Vps67 family protein | 7 | 0.5  1.2  1.8  2.2 (x2)  2.5 (x2) | 3 | 2.5 (x3) | 1 | 2.5 |
| PGSC0003DMT400018414 | Phosphoglycerate mutase | 3 | 0.8  1.5  2.0 | 14 | 0.8  1.2 (x6)  1.5  1.8  2.0 (x3)  2.5 (x2) | 1 | 2.5 |
| PGSC0003DMT400072865 | protein kinase splA | 3 | 2.0  2.2 (x2) | 3 | 2.0  2.2 (x2) | 1 | 2.0 |
| PGSC0003DMT400073246 | aquaporin NIP1-1 | 5 | 2.0 (x2)  2.2  2.5 (x2) | 7 | 1.8 (x2)  2.0 (x2)  2.2  2.5 (x2) | 1 | 2.5 |
| PGSC0003DMT400074377 | Solanum tuberosum Hsp90-2 | 2 | 2.2  2.5 | 3 | 2.2 (x2)  2.5 | 1 | 2.2 |
| PGSC0003DMT400074517 | beta-1,3-galactosyltransferase 2 | 5 | 1.5  2.2 (x2)  2.5 (x2) | 2 | 2.5 (x2) | 1 | 2.2 |

PVY *Potato virus* Y, vsiRNA virus derived small interfering RNA
